# An FXPRLamide Neuropeptide Induces Seasonal Reproductive Polyphenism Underlying a Life-History Tradeoff in the Tussock Moth

**DOI:** 10.1371/journal.pone.0024213

**Published:** 2011-08-26

**Authors:** Hiroshi Uehara, Yukiko Senoh, Kyohei Yoneda, Yoshiomi Kato, Kunihiro Shiomi

**Affiliations:** 1 Faculty of Textile Science and Technology, Shinshu University, Ueda, Nagano, Japan; 2 Department of Life Science, International Christian University, Mitaka, Tokyo, Japan; University of Otago, New Zealand

## Abstract

The white spotted tussock moth, *Orgyia thyellina*, is a typical insect that exhibits seasonal polyphenisms in morphological, physiological, and behavioral traits, including a life-history tradeoff known as oogenesis-flight syndrome. However, the developmental processes and molecular mechanisms that mediate developmental plasticity, including life-history tradeoff, remain largely unknown. To analyze the molecular mechanisms involved in reproductive polyphenism, including the diapause induction, we first cloned and characterized the diapause hormone-pheromone biosynthesis activating neuropeptide (*DH-PBAN*) cDNA encoding the five Phe-X-Pro-Arg-Leu-NH_2_ (FXPRLa) neuropeptides: DH, PBAN, and α-, β-, and γ-SGNPs (subesophageal ganglion neuropeptides). This gene is expressed in neurosecretory cells within the subesophageal ganglion whose axonal projections reach the neurohemal organ, the corpus cardiacum, suggesting that the DH neuroendocrine system is conserved in Lepidoptera. By injection of chemically synthetic DH and anti-FXPRLa antibody into female pupae, we revealed that not only does the *Orgyia* DH induce embryonic diapause, but also that this neuropeptide induces seasonal polyphenism, participating in the hypertrophy of follicles and ovaries. In addition, the other four FXPRLa also induced embryonic diapause in *O. thyellina*, but not in *Bombyx mori*. This is the first study showing that a neuropeptide has a pleiotropic effect in seasonal reproductive polyphenism to accomplish seasonal adaptation. We also show that a novel factor (i.e., the DH neuropeptide) acts as an important inducer of seasonal polyphenism underlying a life-history tradeoff. Furthermore, we speculate that there must be evolutionary conservation and diversification in the neuroendocrine systems of two lepidopteran genera, *Orgyia* and *Bombyx*, in order to facilitate the evolution of coregulated life-history traits and tradeoffs.

## Introduction

**Figure 1:**
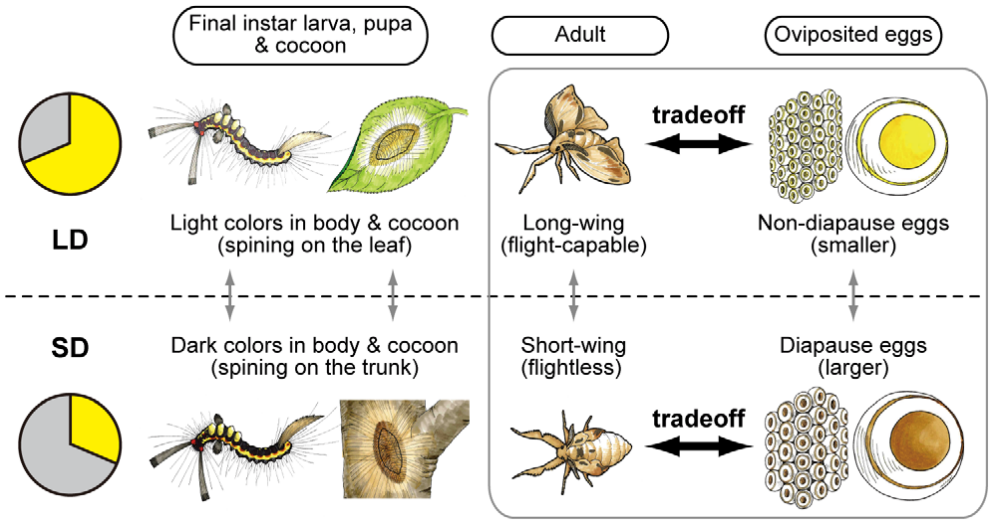
Seasonal polyphenism in the white spotted tussock moth, *Orgyia thyellina*. *Orgyia* exhibits seasonal changes in various morphological, physiological, and behavioral traits, which are determined by the photoperiod during the late larval stages from 4^th^ to 5^th^ instar larvae. In the long-day condition (LD), the larval integument becomes light colored in the final instar larva (6^th^ instar) as does the pupae of the females and their cocoons. The larval integuments and cocoons are darkly colored under short-day conditions (SD). In the adult stage, LD females are flight-capable long-winged morphs, but SD females are flightless short-winged morphs. Furthermore, LD female lay non-diapause eggs, whereas the SD female lay diapause eggs, which are arrested in early embryonic development. These diapause eggs are heavier in weight, larger in size, and much thicker in the chorion than non-diapause eggs [3], [4].

A seasonal polyphenism is a developmental phenotypic plasticity that has evolved for seasonal adaptation, and consists of the differential expression of alternative phenotypes from a single genotype depending on environmental conditions, including the photoperiod, temperature, and nutrition [1], [2]. The white-spotted tussock moth, *Orgyia thyellina*, which belongs to Lymantriidae, has two or three generation per year, and pass winter season as diapause egg. Further, only females of this moth, but not males, exhibits seasonal polyphenism with various phenotypes of morphological, physiological, and behavioral traits in response to photoperiod (Fig. 1) [3], [4]. This sexual difference in photoperiodic response is very unique among many moth species because the sexual dimorphism of other moths is genetically determined [5], [6], [7], [8]. In the adult stage, flight-capable, long-winged females of *O. thyellina* are produced from larvae reared at a long photoperiod, while flightless, short-winged females are formed at a short photoperiod. Females emerging in the summer are of the normal, long-winged morph, but those in the autumn are of the short-winged morph. The summer long-winged female lays non-diapause eggs, whereas the autumnal short-winged female lays diapause eggs, which arrest at early embryonic stage [9]. In addition, various reproductive polyphenisms are exhibited in egg size, eggshell thickness, ovarian weight, and egg number. The diapause eggs are heavier in weight, larger in size, and much thicker in the chorion than the non-diapause eggs, presumably providing richer reserves and a more protective chorion to enhance the adaptation to low temperatures in the overwintering state [3].

In general, long-winged and short-winged morphs have different energetic profiles reflecting their different propensities for migration *versus* reproduction [10]. The polyphenism consists of a flight-capable morph that delays reproduction, and a flightless morph that exhibits substantially elevated fecundity; this is a life-history tradeoff called the “oogenesis-flight syndrome” in various insects [10], [11]. These life-history traits alter the energy allocation of limited internal resources to maximize reproductive success [12], [13], [14]. However, the developmental processes and molecular mechanisms that are integrated with environmental stimuli for seasonal polyphenisms and tradeoff strategies are largely unknown, although presumably the developmental plasticity of a life-history trait has evolved to integrate signals from the environment into normal developmental processes through transcriptional and/or neuroendocrine regulators [2].

The silkworm, *Bombyx mori* (Bombycidae) is a typical insect entering diapause at an early embryonic stage, similar to *Orgyia* as described above [15]. Research on the diapause mechanisms of the silkworm has contributed to the understanding of insect neuroendocrinology and to the technical development of the sericultural industry [16]. Diapause hormone (DH) is a 24 aa peptide amide belonging the Phe-X-Pro-Arg-Leu-NH_2_ (FXPRL amide; FXPRLa) neuropeptide family, which is responsible for embryonic diapause, and functions by acting on a G protein-coupled receptor in the developing ovaries during pupal-adult development in females [16], [17]. The DH*-*pheromone biosynthesis activating neuropeptide (*DH-PBAN*) gene encodes a polypeptide precursor consisting of five FXPRLa neuropeptides: DH, PBAN, and α-, β-, and γ-SGNPs in various insects, including *B. mori* [18], [19], which is exclusively expressed in eight pairs of neurosecretory cells (DH-PBAN-producing neurosecretory cells; DHPCs) located within the subesophageal ganglion (SG) [18], [20], [21].

In this study, we cloned and characterized the *Orgyia DH-PBAN* cDNA, and showed that *Orgyia* DH has a pleiotropic effect in the seasonal reproductive polyphenism, including diapause induction, which may be orchestrated via several signaling pathways to integrate various traits to accomplish the seasonal adaptation. These are the first results to show that a novel factor (i.e., the DH neuropeptide) acts as an important inducer of seasonal polyphenism underlying a life-history tradeoff. Furthermore, we speculate that there must be evolutionary conservation and diversification in the neuroendocrine systems of two lepidopteran genera, *Orgyia* and *Bombyx*, in order to facilitate the evolution of coregulated life-history traits and tradeoffs.

## Results

### Characterization of the *Orgyia thyellina DH-PBAN* cDNA

**Figure 2:**
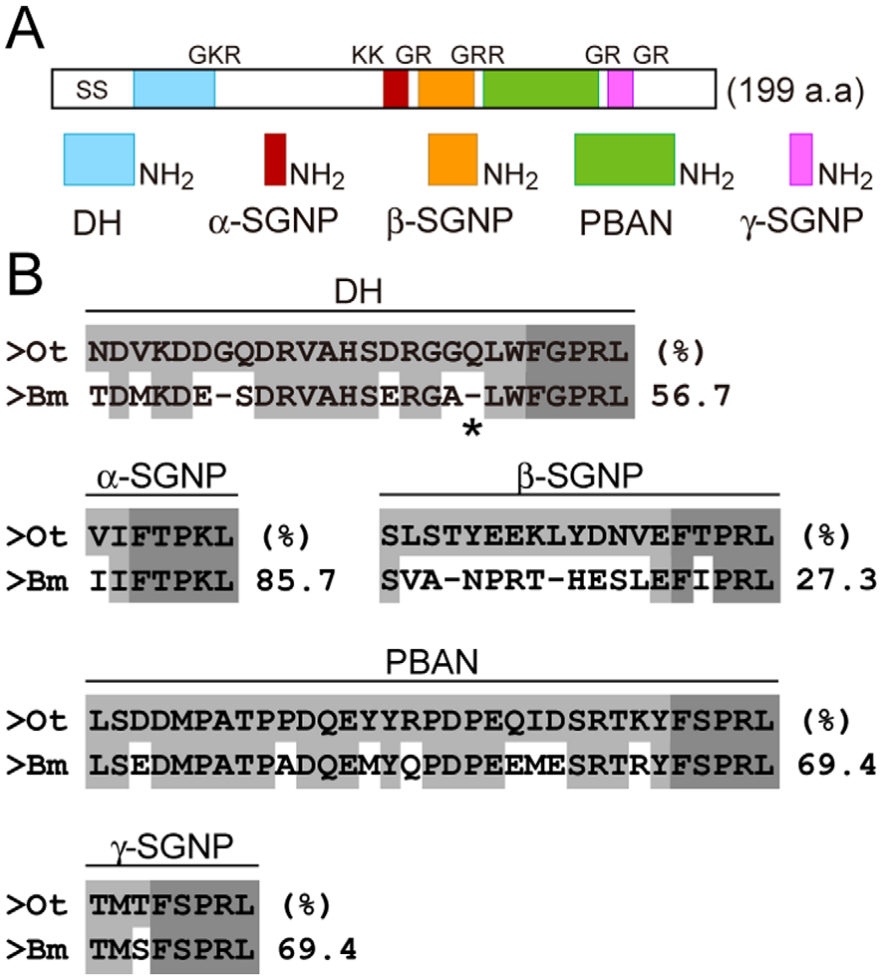
Schematic drawing of the DH-PBAN precursor polyprotein in *Orgyia*. A; *DH-PBAN* cDNA encoding pre-prohormone consisting of 199 amino acids. It seems to undergo post-translational processing via a series of enzymatic steps that cleave and further modify by amidation the GKR, KK, GRR, and 3 GR sequences at the C-terminal amino acid of the intermediate peptide substrates to yield the signal sequence (SS), DH, α-, β-, and γ-SGNP, and PBAN, similar to other Lepidopteran DH-PBAN precursor polyproteins. B; Alignment of *Orygia* DH-PBAN with *Bombyx* DH-PBAN. Conserved amino acids are indicated with shadow boxes; highly conserved amino acids in FXPRLa sequences are indicated with dark shadow boxes. The percentages of identical amino acids is represented on the right side of the peptide sequences. A glutamine residue at position 19 in *Orygia* DH is shown by an asterisk (*).

The *DH-PBAN* cDNA had already been cloned in various insect species, including the Lepidoptera [19], [22]. We cloned the *Orgyia thyellina DH-PBAN* (*OtDH-PBAN*) cDNA from brain-SG complex mRNA using a PCR-based strategy. By aligning the deduced amino acid sequence with the DH-PBAN precursor polyprotein from various insects, we found highly similar characteristics of those precursor polyproteins, especially in the Lepidoptera (data not shown). The *DH-PBAN* cDNAs were highly conserved in all five encoded FXPRLa neuropeptides, including those of *Bombyx* (Fig. 2) [19]. These seemed to include processing sites for the molecular maturation of DH and other FXPRLa neuropeptides through tryptic cleavage and amidation of the GKR, KK, GRR, and 3 GR sequences, as well as a signal peptide cleavage site (Fig. 2A) [18]. Compared with that in *Bombyx*, the C-terminal amino acid sequence, FXPRLa, was completely conserved in each peptide (Fig. 2B). In addition, a glutamine residue at position 19 in DH was inserted in *Orgyia*, although this insertion was not found in other insect species (Fig. 2B). Thus, we first identified the *DH-PBAN* cDNA in Lymantriidae insects, and found that it was highly conserved in other species.

### Developmental expression profiles of *DH-PBAN*

**Figure 3:**
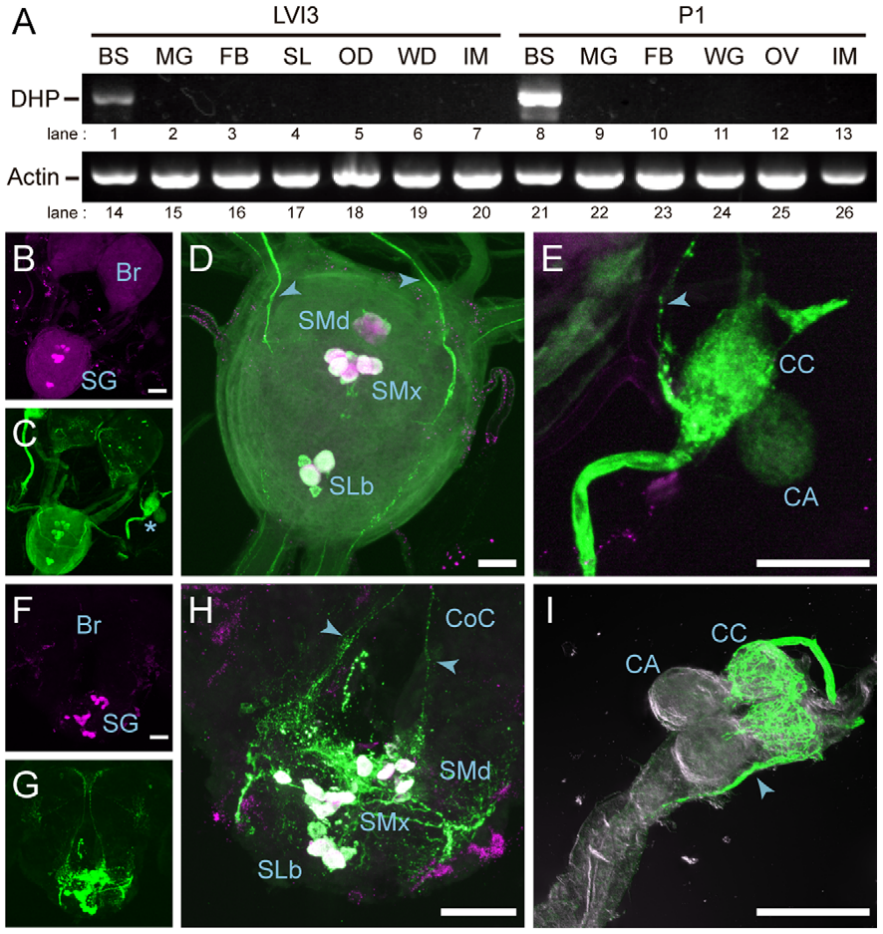
Developmental expression profiles of *OtDH-PBAN*. A; RT-PCR analysis was performed on day 3 in sixth instar larvae (LVI3) as well as on day 1 in pupae (P1). Levels of DH-PBAN (DHP, lanes 1–13) and actin (lanes 14–26) mRNAs were examined. BS, brain-subesophageal ganglion complex; MG, midgut; FB, fat body; SL, silk gland; OD, ovarian disc; WD, wing disc; IM, integument and muscle; WG, wing; OV, ovary. Whole-mount *in situ* hybridization was performed in larval (B) and pupal (F) brain-SG complexes by using antisense RNA of *OtDH-PBAN* as a probe. Using anti-FXPRLa antibody, immunohistochemistry was performed in larvae (C) and pupae (G) stages. Magnified double stained images are shown in magenta (DH-PBAN RNA) and green (anti-FXPRLa) in larval (D) and pupal (H) SG, and larval corpus cardiacum, CC (E). Immunostaining was performed in pupal CC (I). FXPRLa immunoreactive somata were detected in three neuromeres, mandibular cells (SMd), maxillary cells (SMx), and labial cells (SLb), located along the ventral midline. The projective axons (arrowhead) from these somata run into the CC via the circumesophageal connective (CoC). Scale bar = 10 µm.

We looked at the expression of *OtDH-PBAN* in various tissues during larval-pupal and pupal-adult development using RT-PCR analysis. *OtDH-PBAN* mRNA was exclusively expressed in the brain-SG complex during post-embryonic development (Fig. 3A, lanes 1 and 8), although *actin* mRNA was expressed ubiquitously throughout post-embryonic development (Fig. 3A, lanes 14–26). Next, we examined the localization of *OtDH-PBAN* in the central nervous system by whole-mount *in situ* hybridization and immunohistochemistry (Figs. 3B–I). The intensity signal was detected in large somata along the ventral midline within the larval and pupal SG, respectively, in *in situ* hybridization (Fig. 3B and F). Furthermore, immunohistochemical staining with an anti-FXPRLa antibody identified somata in the SGs of both larval and pupal stages (Fig. 3C and G) whose immunofluorescence overlapped with the HNPP/FastRed TR fluorescences of the *DH-PBAN* mRNA probe (Fig. 3D and H). We found that the arrangements of these neuromeres were similar to DHPCs conserved among insect species, which contain three neuromeres, four mandibular cells (SMd), six maxillary cells (SMx), and two labial cells (SLb) located along the ventral midline [23], [24], [25]. Using an anti-FXPRLa antibody, the axons projecting from the DHPCs extended to the circumesophageal connective (Fig. 3H), and their axonal projections reached the neurohemal organ corpus cardiacum (CC) in the larval and pupal SG, respectively (Fig. 3E and I). Therefore, we concluded that *OtDH-PBAN* is expressed in the DHPCs of SG, and the FXPRLa peptides are transported into the CC where they are released into the hemolymph, and that these neuroendocrine processes conserved in other lepidopteran insects.

### Effects of *Orgyia* DH on diapause induction and its metabolism

**Figure 4:**
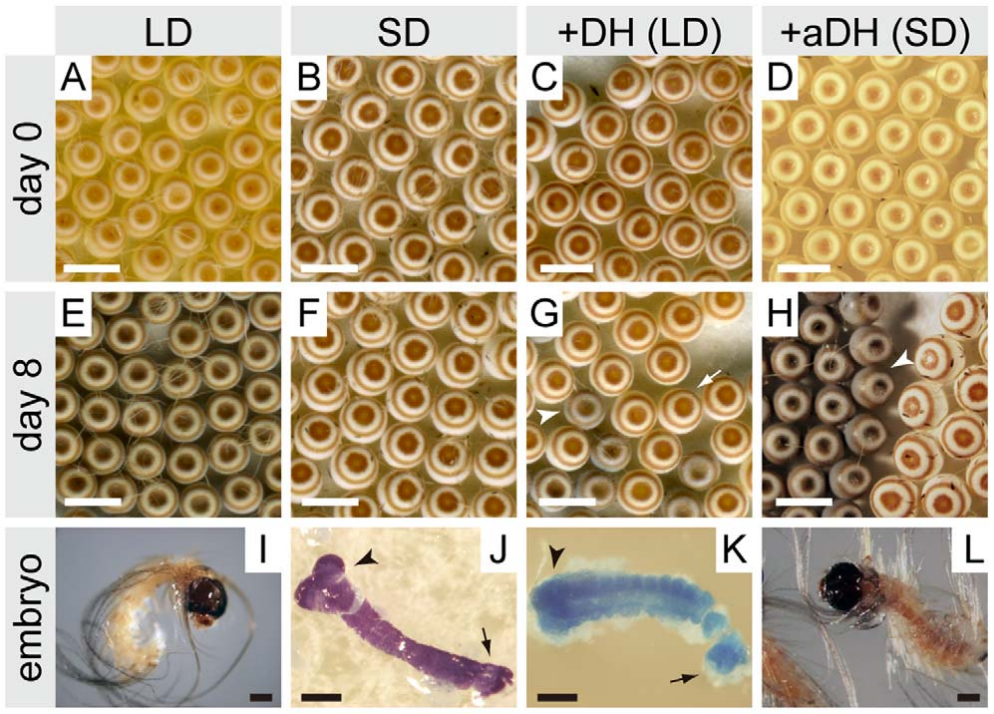
Morphology of *Orgyia* eggs. Eggs laid just after oviposition (A–D), 8 days after oviposition (E–H), and embryos (I–L). LD and SD show eggs (A, E and B, F) and embryos (I and J) oviposited from long-day and short-day conditions, respectively. +DH (LD) and +aDH (SD) show eggs (C, G and D, H) and embryos (K and L) oviposited from pupae injected with DH and anti-FXPRLa, respectively. The white arrow and arrowhead indicate diapause and non-diapause eggs, respectively. The black arrow and arrowheads indicate talson and cephalic lobes, respectively. Scale bar = 5 mm.

**Figure 5:**
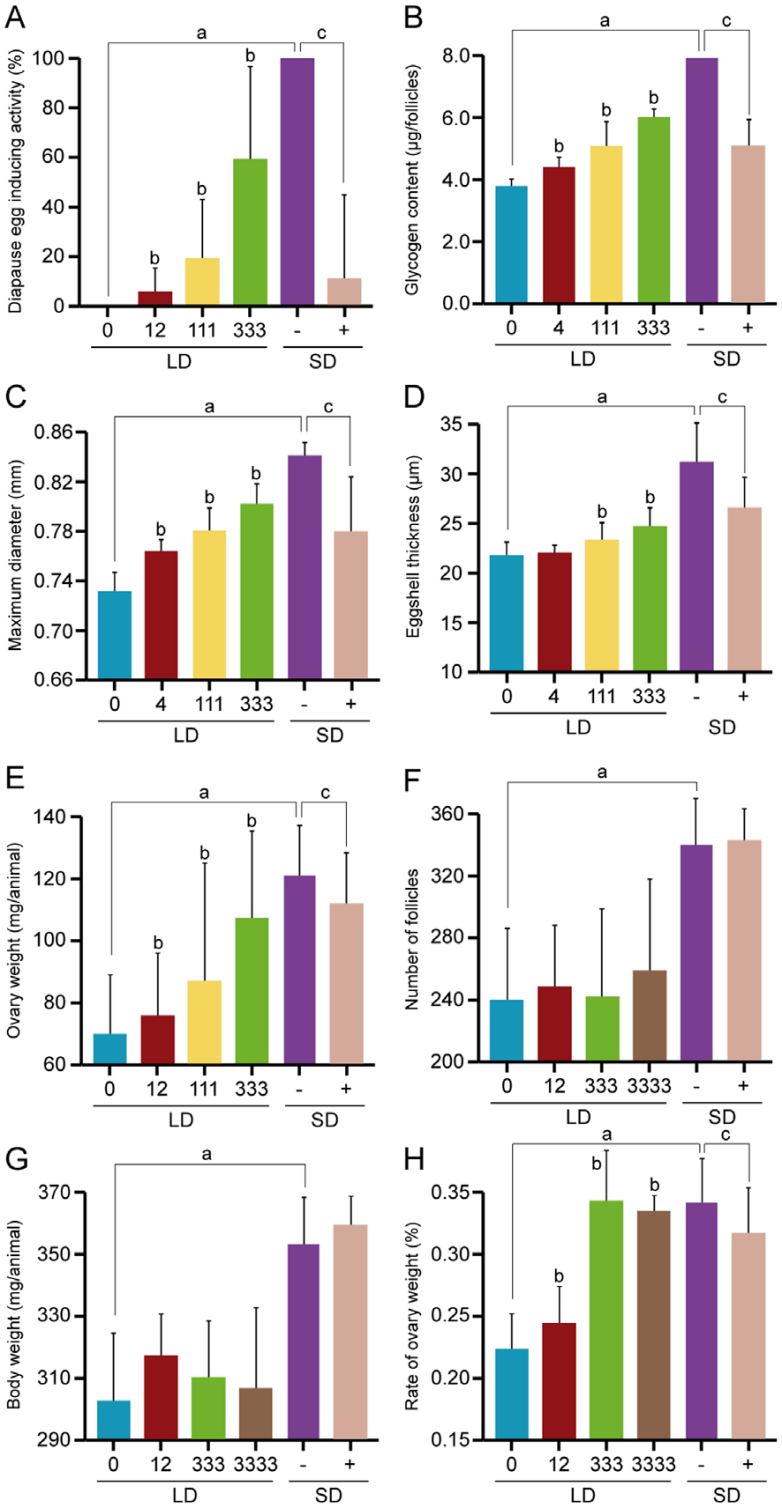
Effects of *Orgyia* DH on diapause and oogenesis involved in seasonal polyphenism. The *Orgyia* DH was injected into LD-pupae at various doses, and analyzed for diapause egg inducing activity (A), glycogen content (B), maximum diameter of follicles (C), eggshell thickness (D), ovary weight (E), number of follicles (F), body weight (G), and rate of ovarian weight (H). Furthermore, anti-FXPRLa serum (+) and pre-immuno serum (−) were injected into SD-pupae to examine the DH functions as described in the text. Each bar represents the mean value of 10 samples ± SD. Lower-case letters indicate statistically significant differences at the 5% level in DH-injected animals at 0 pmol in LD *vs.* pre-immuno serum-injected animals in SD (a), DH-injected animals at 0 pmol in LD *vs.* DH-injected animals at various doses (b), and pre-immuno serum-injected animals in SD *vs.* anti-FXPRLa-injected animals (c).

To determine whether *Orgyia* DH induces embryonic diapause, we injected chemically synthetic DH into pupae kept under long-day condition (LD; non-diapause type) (Fig. 4). When DH was injected into female pupae at 333 pmol/pupa, oviposited eggs took on a dark brown color (Fig. 4C), similar to diapause eggs induced by short-day condition (SD) (Fig. 4B), and were bigger than non-diapause eggs induced by LD (Fig. 4A). At 8 days after oviposition, non-diapause eggs took on pigmentation in the head and body of developing embryos (Fig. 4E and I). In other words, some of the eggs oviposited from DH-injected pupae had the appearance of diapause eggs (Fig. 4G, arrow) obtained by SD (Fig. 4F), although some of the eggs took on pigmentation in the head and body of developing embryos similar to non-diapause eggs (Fig. 4G, arrowhead). In SD animals, we observed that the embryos were arrested and entered diapause in the early embryonic stage, with cephalic lobe and talson formation (Fig. 4J). We also observed variation in diapause stages in DH-injected animals, not only in cephalic lobe and talson formation similar to SD animals, but also in segmentation of the mesoderm in early embryonic development in *Orgyia* (Fig. 4K). These diapause eggs never hatched, even after a year kept at 25°C. However, when the eggs were kept at 5°C for 3 months starting from 7 days after oviposition, and then returned to 25°C, larvae hatched within 10 days (data not shown). Therefore, we categorized these eggs injected with DH as diapause the same as those induced by SD. Next, we injected various amounts of *Orgyia* DH into LD-types. DH caused a dose-dependent increase in the percentage of diapause eggs in a range of 12–333 pmol/pupa (Fig. 5A). The activity was saturated at more than 333 pmol/pupa, and the half-maximal dose was estimated to be 111 pmol/pupa.

In addition to DH injection, we examined whether the injection of anti-FXPRLa antibody affects diapause induction. We injected anti-FXPRLa serum 1 day after pupation in SD-pupae, and observed the diapause nature of the eggs (Fig. 4D, H, and L). Although pre-immune serum had no effect on the diapause status, so that all eggs entered diapause as did the non-treated controls (Fig. 5A, −), injection of anti-FXPRLa caused non-diapause eggs to become light yellow and smaller than diapause eggs in most of the egg batch just after oviposition (Fig. 4D and 5A, +). With advancing embryogenesis, non-diapause eggs took on pigmentation in the head and body of developing embryos (Fig. 4H). The larvae were hatched within 10 days (Fig. 4L). Consequently, these results suggest that the anti-FXPRLa serum acts in the hemolymph to inactivate DH through immunoneutralization.

In general, insects accumulate reserves prior to diapause. Sufficient reserves must be sequestered to both survive the diapause period and enable postdiapause development [26]. In *Bombyx*, diapause eggs are associated with qualitative and quantitative shifts in glycogen metabolism, which accumulate larger glycogen reserves than their nondiapausing counterparts [15]. Therefore, we investigated whether the *Orgyia* DH affects glycogen content in the ovary. The *Orgyia* DH caused an increase in glycogen content in ovaries in a dose-dependent manner, and the injection of anti-FXPRLa suppressed that accumulation (Fig. 5B). Collectively, we demonstrated that the neuropeptide DH induced not only embryonic diapause, but also shifts to diapause metabolism in *Orgyia*.

### Effects of *Orgyia* DH on oogenesis involved in seasonal polyphenism

We further examined whether DH induces various traits in seasonal polyphenism in oogenesis, although none of those changes are observed in *Bombyx*. First, we observed various traits of oogenesis in LD- and SD-pupae. The eggs of SD-animals were larger, heavier, had a thicker chorion, had a greater number of follicles, and were heavier in the whole body compared to those of LD-animals (Fig. 5C–G). Next, we investigated whether the *Orgyia* DH affected those phenotypes. *Orgyia* DH caused an increase in the maximum diameter, eggshell thickness, and ovary weight in a dose-dependent manner (Fig. 5C–E), although there were differences in response for each trait (Fig. 5D *vs*. A–C and E). In addition, injection of the anti-FXPRLa into SD animals induced dwarfness in eggs including smaller size, thinner maximum eggshell thickness, and lighter ovaries similar to non-diapause eggs (Fig. 5C–E). However, no changes were observed in the number of follicles and body weight by *Orgyia* DH and antiserum injections (Fig. 5F and G).

Consequently, *Orgyia* DH acts on the ovarian mass (Fig. 5E and H). Interestingly, although the ovarian masses of LD- and SD-pupae were 20% and 35%, respectively (Fig. 5H), DH-injected animals in LD-pupae were similar to SD animals in terms of ovary mass and whole body weight irrespective of the injection of excess amounts of DH. Thus, *Orgyia* DH induced various traits involved in the seasonal reproductive polyphenism underlying the life-history tradeoff known as oogenesis-flight syndrome.

### Effects of other *Orgyia* FXPRLa on diapause induction in both *Orgyia* and *Bombyx*

**Figure 6:**
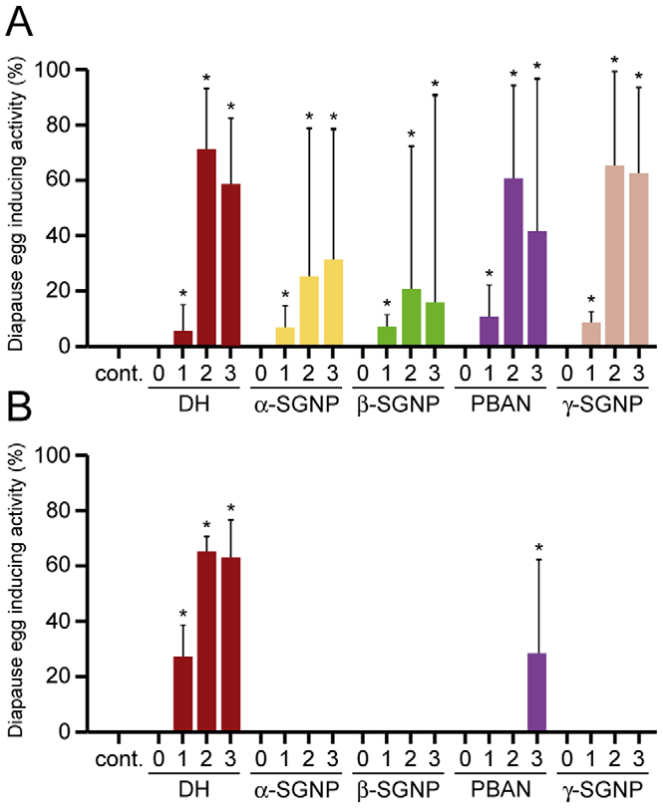
Effects of other *Orgyia* FXPRLa on diapause induction in both *Orgyia* and *Bombyx*. The *Orgyia* DH, α-, β-, and γ-SGNPs, and PBAN were injected into LD-pupae of *Orgyia* (A) and the non-diapause type of *Bombyx* (B) at various doses [3 (0), 33 (1), 333 (2), and 3333 (3) pmol/pupa], and subjected to analysis of diapause egg inducing activity. Each bar represents the mean value of 20 samples ± SD. Asterisks indicate statistically significant differences at the 5% level.

Next, we examined whether other FXPRLa (α-, β-, γ-SGNPs, and PBAN) induced embryonic diapause with a similar dose-response as DH (Fig. 6). We found that all FXPRLa had diapause egg inducing activity, which increased dose-dependently in a range of 33–333 pmol/pupa similar to *Orgyia* DH in *Orgyia* (Fig. 6A). These diapause eggs inducing activities were saturated at more than 333 pmol/pupa. Furthermore, we tested whether each *Orgyia* FXPRLa also induced embryonic diapause in *Bombyx* (Fig. 6B). Interestingly, only *Orgyia* DH had a similar effect with the dose used in *Orgyia*, but PBAN showed diapause egg inducing activity only at 10 times amount of DH. No activity was found in α-, β-, or γ-SGNPs in this experiment (Fig. 6B). These results showed that the diapause inducing activity of *Orgyia* FXPRLa is different between *Orgyia* and *Bombyx*, suggesting that the mechanism of hormonal reception is different in the two species, given that DH and the other FXPRLa have similar structures in both species.

## Discussion

Previously, *Bombyx* DH was known as the only hormone that induces diapause among known FXPRLa neuropeptides [16]. Furthermore, *Bombyx* DH stimulates the transcription of the trehalase gene in ovaries, and thereby increases trehalase activity which facilitates higher accumulation of glycogen in eggs, a prerequisite for diapause initiation [27], [28] that sequesters sufficient reserves for survival of the diapause period and postdiapause development [26]. We found that *Orgyia* DH also acts as a diapause inducer during embryogenesis causing development to be arrested, and enhances the glycogen content, which seems to affect the conserved neuroendocrine system in both *Bombyx* and *Orgyia*. We think it is most likely that in SD-type morphs, transportation of DH from the DHPCs somata is actively modified in response to the photoperiod during late larval stages and results in active release into the hemolymph during the pupal stages, likely as in *Bombyx* [29]. Thus, we showed that the DH acts as a diapause inducer beyond the families Bombycidae and Lymantriidae in lepidopteran insects.

DH and other FXPRLa are multifunctional peptides that play a conserved role in the coordination of life-cycles among and within species. DH acts not only during diapause induction in *Bombyx* [16], but is also thought to function during ecdysteroidgenesis [30]. Furthermore, the neuropeptides of this family are involved in pheromone biosynthesis, cuticular tanning, myostimulation, pupariation behavior, and the termination of pupal diapause in several lepidopteran insects [31], [32], [33], [34], [35]. In addition to diapause induction, *Orgyia* DH affects various traits in ovarian growth and maturation such as follicle size, eggshell thickness, and ovarian mass in different dose-dependent manner, although none of these changes are observed in *Bombyx*. These results suggest that there are several signaling pathways involved in the induction of these various traits. In general, the developing follicle processes the transition among broad developmental periods, which is mediated by hormones such as ecdysteroid and a sesquiterpenoid lipid-like hormone, JH, in insects [36]. In *Bombyx*, ecdysteroid was shown to be essential and sufficient for ovarian development through the activation of the ecdysone regulatory cascade [37], [38]. Furthermore, the injection of large amounts of 20-hydroxyecdysone (20E) into normal female pupae of *Bombyx* alters egg production, so that these eggs are larger and heavier than normal eggs [39], suggesting that the ecdysteroid may induce an increase in the number of oocytes by functional activation of nurse cells, to accelerate the growth of oocytes accompanied by enhanced accumulation of yolk proteins and accelerated choriogenesis. Taken together, this suggests that *Orgyia* DH may induce egg polyphenism directly and/or indirectly via several signaling pathways, including 20E signaling, which may function via ecdysteroidgenesis as in *Bombyx* [30]. Thus, this is the first evidence suggesting that a neuropeptide (i.e., *Orgyia* DH) alone has a pleiotropic effect in seasonal reproductive polyphenims, which may be orchestrated via several signaling pathways to integrate various traits, since these polyphenisms are considered to provide richer reserves and more protective chorion to enhance adaptation to low temperatures.

Neuroendocrine systems are known to coordinate the integrated expression of multiple life-history traits across environmental conditions as developmental phenotypic plasticity, which mediates pleiotropy, life-history correlations, and tradeoffs in organisms as diverse as lizards, birds, insects, and echinoderms [13], [40]. In insects, JH and ecdysteroid are well known to be important mediators of life-history tradeoffs [13], [14], [41]. Indeed, it has been suggested that JH and ecdysteroid are involved not only in the tradeoff between flight capability and reproduction in wing-dimorphic crickets, *Gryllus firmus* [42], but also in tradeoffs in various insects including *Drosophila*, butterflies, and beetles [14], [43]. Furthermore, it is known that JH alters lipid metabolism that contributes significantly to the tradeoff in *G*. *firmus* [44]. We have also confirmed that various traits with respect to ovarian and embryonic development change between long-winged and short-winged morphs in *Orgyia* (Fig. 1) [3], [4]. Furthermore, it has been observed that the flight muscles become extended and increase in thickness in all long-winged morphs, while the flight muscles remain thin in short-winged morphs (Y. K, unpublished results). Thus, *Orgyia* exhibits an extreme seasonal polyphenism resulting in a tradeoff between flight capability and reproduction, resulting in differential allocation of internal reserves to ovarian growth versus somatic growth, maintenance, or storage. These are the first results to show that a factor (i.e., DH neuropeptide) other than a sesquiterpenoid lipid-like hormone or steroid hormone (i.e., JH and ecdysteroid) can act as an important inducer of developmental phenotypic plasticity underlying a life-history tradeoff, although it is still unknown what the mechanism of energy allocation is and its involvement in wing development.

Tight control is necessary to produce an animal in which each organ is of an appropriate size relative to the whole body. Different organs typically grow at different rates in developing animals, a phenomenon called “allometry” [45], [46]. The flexibility of allometry is necessary to produce an animal in which the whole body is of an appropriate size relative to environmental conditions such as nutrition, temperature, and population density [47], [48]. Recent studies have indicated that the insulin-signaling pathway controls body and organ size in *Drosophila* and most metazoans by signaling nutritional conditions to the growing organs [49], [50]. In this study, the rates of ovarian mass to body increased to accompany the increase in ovarian weight by DH injection, so that LD animals injected with DH, even in excess doses, shifted to become similar to SD animals. Therefore, we speculate that there is inherent allometry in ovarian mass in both LD- and SD-types, which determine the photoperiodism. Additionally, the DH-signaling pathway might be involved in the regulation of ovary-body allometry, although it is unknown whether other signals are involved, such as the insulin-signaling pathway.

It has been suggested that variations in endocrine mechanisms may be an important substrate for the evolution of coregulated life-history traits and tradeoffs [14]. Evolutionary modifications of the hormone response may be facilitated by its modular structure [40]. In a previous study, *Bombyx* DH was found to be solely responsible for embryonic diapause and functions by acting on a specific G protein-coupled receptor in developing ovaries, as other FXPRLa never act at similar doses to DH [16], [17]. Likewise, *Orgyia* FXPRLa, except for DH, never induce embryonic diapause at similar doses of DH in *Bombyx*. Interestingly, in the present study we found that *Orgyia* FXPRLa functions in diapause induction in a similar dose-dependent manner to DH in *Orgyia*. This result suggests that the structure and/or densities of receptor protein(s) may be different with evolutionary diversification in the two species. Variation in the expression of the receptor protein(s) of the *Orgyia* FXPRLa family may occur in the two species, so that those differences may promote the hormonal pleiotropy in the polyphenism. Therefore, the present study may provide not only clues for solving the molecular mechanisms of life-history evolution, but also provide new insights into comparative neuroendocrinology in insects.

## Materials and Methods

### Insects

Eggs of the white spotted tussock moth, *Orgyia thyellina*, were collected at an apple orchard in the Nagano Fruit Tree Experiment Station (Suzaka, Japan). The hatched larvae were reared on an artificial diet (Silkmate-2S, Nosan Co., Yokohama, Japan) from first instar to third instar, and fresh apple leaves from fourth instar to final instar (sixth instar in females and fifth instar in males) at 22°C under LD (16-h light/8-h dark cycle; 16L:8D) or SD (8L:16D). Pupae were collected within the day just after pupation (referred to as day 0) to synchronize their subsequent development. Pupae were kept at 25°C to allow adult development.

The polyvoltine (N4) strain of *Bombyx mori* was used in this study. Eggs were incubated at 25°C under continuous darkness. Larvae were reared on an artificial diet (Kuwano-hana, JA Zennoh Gunma, Gunma, Japan). During larval stages, silkworms were reared at 25 to 27°C under a 12L:12D. Pupae were kept at 25°C to allow adult development. N4 moths laid only non-diapause eggs genetically, so that eggs never entered diapause.

### cDNA cloning

Poly (A)^+^ RNA was directly purified from the brain-SG complexes of day 0 pupae of *Orgyia* using Dynabeads Oligo (dT)_25_ (Dynal Biotech LLC., Brown Deer, WI, USA). To amplify the *DH-PBAN* cDNA, RT-PCR was performed using degenerate primers based on sequences of the *DH-PBAN* cDNA cloned from various insects: 5′-TGGTTCGGYCCCMGRCT-3′ (forward) and 5′-GGHGTRGCWGGCAT-3′ (reverse). The full-length sequence (Accession no. AB259122) was determined using a SMART RACE cDNA amplification kit (Clontech, Mountain View, CA, USA). The location of signal peptide cleavage sites in amino acid sequences were predicted by the SignalP 3.0 server (http://www.cbs.dtu.dk/services/SignalP/). Amino acid alignment of FXPRLa neuropeptides was performed by CLUSTALW (1.83) (http://clustalw.ddbj.nig.ac.jp/top-j.html).

The ORF sequence of the *actin* cDNA was amplified using primers based on sequences of the *actin* cDNA cloned from various insects: 5′-ATGTGCGACGARGAAGTTGC-3′ (forward) and 5′-TTAGAAGCACTTCCTGTGNAC-3′ (reverse). The full-length sequence (Accession no. AB283042) was determined using a SMART RACE cDNA amplification kit (Clontech).

### RT-PCR analysis

Various tissues were dissected from day 3 sixth instar larvae and day 1 pupae of females reared in SD. Total RNAs were extracted using the TRIzol reagent (Invitrogen, Carlsbad, CA, USA) and then subjected to Poly (A)^+^ RNA purification using Dynabeads Oligo (dT)_25_ (Dynal Biotech LLC.). Poly (A)^+^ RNA from the brain-SG complex was directly purified using Dynabeads Oligo (dT)_25_. First-strand DNA was synthesized using a SMART RACE amplification kit (Clontech). PCR amplification was carried out on mRNAs for *DH-PBAN* and *actin* genes. The *DH-PBAN* cDNA was amplified from nucleotides +1 to +759, and the *actin* cDNA from +238 to +722. The PCR products were subjected to electrophoresis, and then visualized with ethidium bromide staining.

### *In situ* hybridization and Immunohistochemistry

*In situ* hybridization was performed as described by [51] with some modifications. Brain-SG and retrocerebral, corpus cardiacum (CC)-corpus allatum (CA) complexes were treated for 5 min or 3.5 min with 10 µg/ml proteinase K (Roche) at 37°C on larval or pupal tissues, respectively. The DIG-labeled RNA probes were prepared with a DIG RNA labeling kit (Roche, Mannheim, Germany) using *DH-PBAN* cDNA as a template. *DH-PBAN* cDNA encoding from nucleotides +1 to +759 was amplified by PCR and inserted into the pCR-XL-TOPO vector (Invitrogen) in an antisense direction from the T7 promoter. DIG-labeled RNA was detected with an alkaline phosphatase-conjugated anti-DIG antibody using a DIG nucleic acid detection kit (Roche), and by using the HNPP fluorescent detection set (Roche) as a substrate. The immunoreaction procedures were adapted from [29] by using an anti-FXPRLa polyclonal antibody [25], [29]. HNPP/FastRed TR fluorescence and anti-FXPRLamide immunofluorescent staining were detected using both a Radiance 2000 confocal microscope (Bio-Rad Lab., Hercules, CA, USA) and confocal microscope system A1 (Nikon, Tokyo, Japan). Images were adjusted and assembled in Adobe Photoshop CS3 (Adobe Systems, Inc., San Jose, CA, USA).

### Injection of peptides and antibody

Purified synthetic peptides (DH, α-, β-, γ-SGNP, and PBAN) of >95% purity (HPLC area percentage) were provided from Operon Biotechnologies, KK. (Tokyo, Japan). Each peptide was dissolved in distilled water, and 10 µl solutions of each at various doses were injected into pupae on the day of pupation (day 0) of *Orgyia*, and 4 days after pupation in the N4 strain of *Bombyx*. Anti-FXPRLa antiserum was injected at 10 µl into *Orgyia* pupae on day 0 in accordance with [27].

### Analyses of oogenesis

The ovaries were dissected out with phosphate-buffered saline (PBS) 1 day before eclosion when pupae integuments became white, and the wing color patterns of the adult forms were visualized. The paired ovaries were thoroughly separated into 8 ovarioles, then blotted dry, weighed quickly, and the number of follicles was counted. In each ovariole, 20 successively developing follicles from side of the lateral oviducts were collected to measure the maximum diameter, eggshell thickness, and glycogen content. The maximum diameter and eggshell thickness were measured by scanning on a SZX-12 microscope (Olympus, Tokyo, Japan) with a micrometer. Glycogen was extracted by digesting the homogenate with 30% (^W^/_V_) KOH in a boiling bath for 30 min, and was precipitated with ethanol at 4°C [52], and measured by the phenol-sulfuric acid method [53].

### Observation of embryogenesis and diapause

In both *Orgyia* and *Bombyx*, eclosed female moths were allowed to copulate with males overnight, and then to lay eggs overnight. The laid eggs were kept at 25°C, under which condition the non-diapause eggs hatched on day 7 to 8 after oviposition. Some eggs became light white or yellow in a few days and were recognized as non-fertilized eggs. Diapause eggs were identified as eggs that were colored dark brown on day 3 and remained unhatched at 2 weeks after oviposition, at which time all non-diapause eggs had hatched. Diapause egg inducing activity was estimated by counting the number of diapausing and non-diapausing eggs after the non-diapause eggs hatched, and the results were expressed as a percentage of diapause eggs [54]. The embryos were stained with carbolic thionin solution according to [55]. Eggs were mounted on a slide glass sealed with adhesive tape, and then eggs were sliced with a razor and observed under a microscope.
